# Associations between Aromatase CYP19 rs10046 Polymorphism and Breast Cancer Risk: From a Case-Control to a Meta-Analysis of 20,098 Subjects

**DOI:** 10.1371/journal.pone.0053902

**Published:** 2013-01-16

**Authors:** Begoña Pineda, Miguel Ángel García-Pérez, Antonio Cano, Ana Lluch, Pilar Eroles

**Affiliations:** 1 Institute of Health Research INCLIVA, Valencia, Spain; 2 Department of Genetics, University of Valencia, Valencia, Spain; 3 Department of Pediatrics, Obstetrics and Gynecology, University of Valencia, Valencia, Spain; 4 Department of Haematology and Medical Oncology, University Hospital of Valencia, Valencia, Spain; The University of Texas M. D. Anderson Cancer Center, United States of America

## Abstract

Lifetime exposure to estrogen is a factor that plays an important role in the pathogenesis and progression of breast cancer. Genetic variants in genes of the biosynthesis and metabolism of estrogen have been associated with breast cancer risk. Among them, the *CYP19* gene encodes for aromatase, the enzyme that catalyzes the conversion of androgens to estrogens. The rs10046 polymorphism on the *CYP19* gene has been related to levels of circulating estradiol and to the estradiol/testosterone ratio. To date, epidemiological studies of rs10046 have been performed in different populations with contradictory results. In the present study, we have conducted a case-control analysis (522 cases and 1221 controls) in a Spanish population. Furthermore, we have performed a meta-analysis including 20,098 subjects (7,998 cases and 12,100 controls) to summarize the data available for rs10046 and breast cancer risk. An odds ratio (OR) with a 95% confidence interval (CI) was applied to assess the association. The results of our case-control study show an association between the carriers of at least one C allele (dominant model) and breast cancer risk (OR = 1.29, 95% CI 1.01–1.66, p-value = 0.038). The meta-analysis shows no significant association with breast cancer risk in any of the genetic models tested. The analysis by ethnic subgroups also failed to produce associations. The evaluation of heterogeneity, influence analysis, and publication bias confirms the reliability of the analysis. We can conclude that the rs10046 polymorphism on *CYP19* by itself does not constitute breast cancer risk. We cannot, however, reject the possibility that it could contribute (interact), together with other genetic variants, to modify the circulating levels of estradiol.

## Introduction

In recent decades, breast cancer cases have increased worldwide [1] and today are the main cause of cancer mortality and morbidity in women [2]. Breast cancer is caused by environmental and genetic factors [3]. Two main genes, *BRCA1* and *BRCA2*, are associated with hereditary breast cancer. Sporadic cases of breast cancer, however, may be related to variants in low-penetrance genes such as polymorphisms [4], [5].

Lifetime exposure to estrogen is another factor that plays an important role in breast cancer. It is known that this hormone is involved both in the development of the mammary gland, as well as in the pathogenesis and progression of breast cancer [6]. Based on this, the study of genes related to biosynthesis and the metabolism of estrogen is one way to identify possible candidate genes for breast cancer risk [7]. One of them is the *CYP19* (P450arom) gene. The human *CYP19* gene is located in the chromosome 15q21.2 region and is comprised of a 30 kb coding region [8]. This gene encodes for aromatase, an enzyme whose function is to catalyze the conversion of androgens into estrogens, a reaction known as aromatization. In premenopausal women, the main source of estrogens is the ovaries. Meanwhile, in postmenopausal women, aromatization takes place elsewhere such as in adipose tissue, skin, muscle, and liver cells.

There have been several epidemiological studies of polymorphisms on the *CYP19* gene with the aim of finding associations between genetic variations and breast cancer risk. Some have found an association with an increased risk of breast cancer, such as that of a tetra-nucleotide repeat polymorphism in the intron 4 (TTTA)n [9]. Still, other polymorphisms studied have not shown a clear association with breast cancer risk, thus generating a situation of inconsistent results. This is the case of a C/T single nucleotide polymorphism (SNP) located in the 3′ untranslated region (3′-UTR) of the *CYP19* gene (rs10046). Some studies have linked this polymorphism with breast cancer risk [10], however, others show different results [11], [12], [13]. This discrepancy in results led us to conduct a case-control study of this SNP in a population in Valencia (Spain). Additionally, we performed a meta-analysis of this polymorphism for the first time. It is a powerful tool for overcoming the problems of the small sample size and inadequate statistical power of genetic studies. This approach gives more reliable results than a single case–control study can. The aim was to collect all results published to date about this polymorphism and to obtain conclusive results about their relevance in susceptibility to breast cancer.

## Materials and Methods

### Case-control Study

#### Study population

Association analysis between the rs10046 polymorphism (*CYP19*) and breast cancer disease was performed in a case-control study. The study was performed in a Caucasian Spanish population composed of 522 breast cancer patients and 1221 controls recruited at the Clinic Hospital of Valencia (Spain) with a mean age at diagnosis of 51 years (range 21–89) and 51 years (range 18–86), respectively. The controls were women without malignant pathology recruited at the blood donor bank and women with non-malignant pathology from the menopausal unit of the same hospital. The recruitment of the cases and controls was performed in the same interval of time ±0.5 years. The research protocols were approved by the ethics committee of the Institute of Health Research INCLIVA before the study began. All the participants in the study gave their written informed consent to participate in the study.

#### Genotyping

Genomic DNA was extracted from blood samples using the DNeasy tissue kit from Qiagen (Izasa, Madrid, Spain) or DNA Isolation Kit by MOBIO (Carlsbad, CA, USA) using minor modifications to the manufacturer protocol. A final elution volume of 100 µl was established. DNA quantity was measured by absorbance at 260 nm using a NanoDrop spectrophotometer, and DNA purity was evaluated by measurement of the 260/280 absorbance ratio. DNA samples were stored at −20°C. Genotyping analysis was performed by real-time PCR (5 ng/ul DNA), using the TaqMan SNP Genotyping Assays C___8234731_30 (Applied Biosystems) according to the manufacturer instructions. Thermal cycling and detection was performed on the ABI Prism 7900 using the Sequence Detection Software (Applied Biosystems). The results were analyzed using the allelic discrimination assay program of Sequence Detection Software version 2.4 (Applied Biosystems).

#### Statistical analysis

The SNPs genotype analysis of our study population was done with the SNPstats software [14] (allele and genotype distributions, association test, Hardy-Weinberg Equilibrium (HWE)). SNPstats association was based on binary logistic regression according to the response variable providing odds ratios (ORs), the confidence interval (CI), and the p-values for multiple inheritance models (dominant, recessive, over-dominant co-dominant, and log-additive). The lowest Akaike’s Information Criterion and Bayesian Information Criterion value indicate the best inheritance genetic model for each specific polymorphism.

### Meta-analysis

#### Literature search strategy for identification of the studies

We did a literature search in PubMed, Scopus and EBSCO data bases using the terms “breast cancer and rs10046”, along with additional terms such as “polymorphisms, SNPs and CYP19”, and all possible combinations. The studies for the meta-analysis were selected when they satisfied the following criteria: studies published by March 2012, case-control studies in humans, studies with genotype frequencies or OR data, information about HWE and information about procedure (adjusted or not, subgroups, etc.). In order to search more deeply, we reviewed the references of the selected articles to retrieve data that we could have ignored in the initial search.

#### Data extraction

Information was extracted from the articles by two of the authors following the criteria listed above (B.P. and P.E.). Disagreement was discussed and resolved between the two authors. In the event that a study presented subpopulations, these were taken to be different studies. The same was done for studies composed of a first set and a second independent validation set.

#### Statistical analysis

Raw data from comparable studies were analyzed jointly using likelihood methods. The estimate of association with breast cancer risk was evaluated using the fixed effect method [15] which calculates the ORs and the corresponding 95% CIs for individual studies and the global association. When the test was heterogeneous, the random-effects method [16] was applied. If heterogeneity was not corrected, we performed an influence analysis to determine the study responsible for that variability. Recessive, dominant, co-dominant, additive and over-dominant models were computed.

The analysis of heterogeneity between studies was performed by the Q statistic, with p-values <0.1 indicating significant heterogeneity [17]. We also used the I^2^ statistic to quantify heterogeneity; values of 25% correspond to low heterogeneity, 50% to moderate heterogeneity and 75% to high heterogeneity [18]. The meta-regression was performed with the SPSS package (Statistical Package for the Social Sciences, version 19.0), and a variable was considered a source of heterogeneity when the p-value was significant in the ANOVA analysis. Publication bias was assessed by funnel plots of effect sizes versus standard errors to identify significant asymmetry. Additionally, Egger’s linear regression test [19] and Begg’s test [20] were performed to evaluate the potential bias. The Gleser-Olkin method [21] was used to estimate the number of unpublished studies. Accumulative meta-analysis [22] was assessed by year of publication to evaluate the possible publication bias by time. Statistical analysis of association was done with SPSS, version 19.0.

## Results

### rs10046 Genotype in a Spanish Population

The distribution of the genotype frequencies in this polymorphism within the control group is in agreement with that expected under HWE with a p-value of <0.05. We also observed that the frequencies in this study were similar to those previously reported in the European population described by HapMap (http://hapmap.ncbi.nlm.nih.gov/).

Our results show an association between the rs10046 polymorphism on the *CYP19* gene and breast cancer risk. The carriers of at least one C allele (dominant model) have 1.29 times increased risk of developing breast cancer (95% CI 1.01–1.66, p-value = 0.038) *vs*. non-carriers. The distribution of the rs10046 genotype among cases and controls and risk of breast cancer is listed in Table 1 (codominant and dominant models).

**Table 1:** Genotypic and allelic frequencies of rs10046 and breast cancer risk.

Polymorphism	Genotype	Cases(n = 522)	Controls(n = 1221)	OR (95%CI)^a^	p-value
rs10046	TT	109 (20.9%)	311 (25.5%)	Reference	
*CYP19*	TC	278 (53.3%)	629 (51.5%)	1.26 (0.97–1.64)	0.094
	CC	135 (25.9%)	281 (23.0%)	1.37 (1.02–1.85)	0.038
	TC+CC	413 (79.1%)	910 (74.5%)	1.29 (1.01–1.66)	

### Meta-analysis Study Characteristics

**Figure 1:**
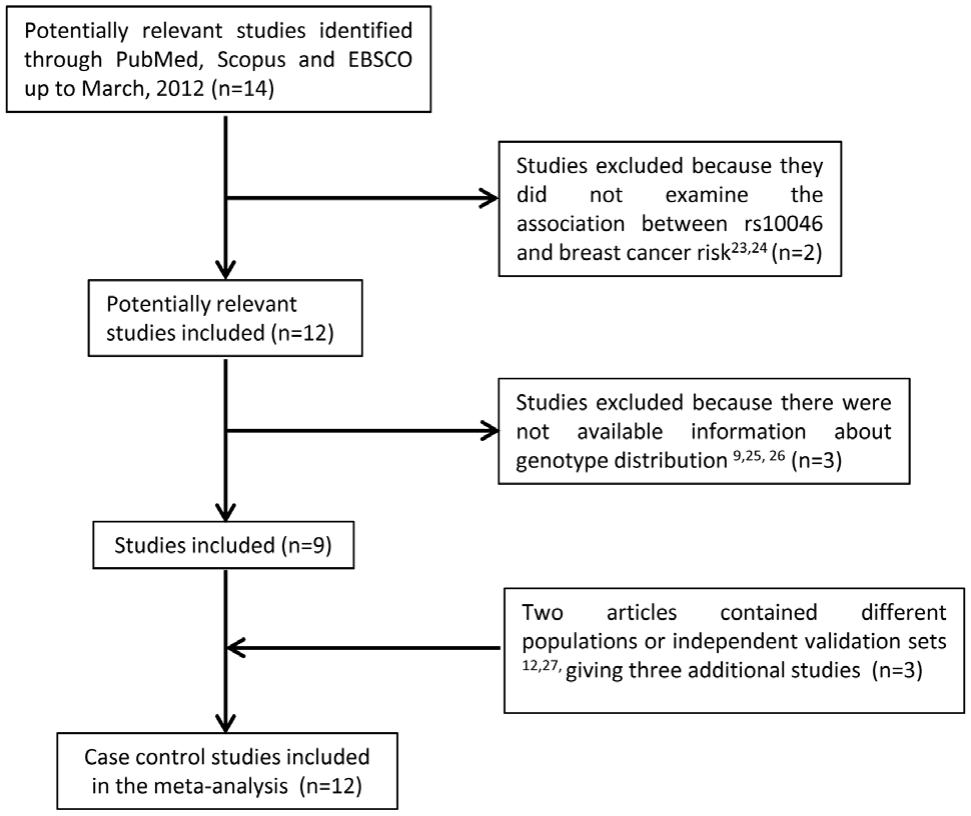
Flow diagram of included/excluded studies.

A total of 14 studies were selected from the bibliography search regarding the association between the rs10046 polymorphism on the aromatase *CYP19* gene and breast cancer. Two of these were excluded for not having been case-control studies [23], [24]. Additionally, of the remaining 12 studies, 3 were excluded for not stating the genotype distribution [9], [25], [26]. Afterwards, the studies presenting different subpopulations according to ethnicity [27] or having a validation set [12] were divided into independent studies. Finally, 12 studies were accepted for the first association analysis using the genotype distribution in cases and controls (Figure 1). Table 2 lists the main characteristics of these studies: first author, publication year, original country, ethnicity, source of controls, genotype distribution of controls and cases, HWE p-value, mean age and menopausal status.

**Table 2:** Main characteristics of the meta-analysis papers and genotype distribution of rs10046.

FIRST AUTHOR	YEAR	COUNTRY	ETHNICITY	SOURCE	GENOTYPE DISTRIBUTION (CONTROLS)					GENOTYPE DISTRIBUTION (CASES)	HWE	MEAN AGE	MENOPAUSAL STATUS
					TT	CT	CC	TT	CT	CC			
Olson	2006	US (Minnesota)	Caucasian (94%)	H	NA						–		
Haiman	2002	US (Nurses’ Health Study)	Caucasian	H	167 (27%)	310 (51%)	134 (22%)	118 (26%)	240 (52%)	103 (22%)	–		
Raskin	2009	Israel (Jews Ashkenazi)	Jews	H	NA						>0.05		
Ralph	2007	USA*	Caucasian	H	883 (26.8%)	1650 (50.1%)	758 (23.1%)	461 (28.1%)	830 (50.6%)	349 (21%)	0.6	cases 9.8(±9.3) controls 49.8(±9.3)	
Ralph (validation)	2007	USA*	Caucasian	H	274 (27.3%)	503 (50.1%)	222 (22.1%)	142 (28.3%)	231 (46%)	129 (25.7%)	>0.05		
Zhang	2008	China	Asian	H	120 (30.8%)	176 (45.1%)	94 (24.1%)	94 (31.3%)	151 (50.3%)	55 (18.3%)	0.43	cases 52.04±11.81 controls 50.41±9.15	cases controls TT CT CC TT CT CC pre 29% 49% 22% 30% 42% 28% post 34% 52% 14% 31% 50% 18%
Kristensen	2000	Norway	Caucasian	HP	53 (23%)	114 (48%)	69 (29%)	146 (31%)	240 (50%)	95 (19%)	>0.05		
Colomer	2008	Spain	Caucasian	H				17 (26%)	29 (45%)	19 (29%)	–		
Fasching	2008	Germany	Caucasian	H				362 (29%)	606 (48.6%)	279 (22.4%)	>0.05		
Dunning	2004	Inglaterra	Caucasian	H	1049 (28.9%)	1773 (48.9%)	808 (22.2%)	739 (28%)	1286 (48.9%)	610 (23.1%)	0.3	cases 22–65 controls 45–74	
Yoshimoto	2011	Japan	Asian	H	60 (21.7%)	120 (43.3%)	97 (35%)	160 (19.3%)	427 (51.7%)	239 (29%)	>0.05		
Garcia-Casado	2010	Spain (Valencia)	Caucasian	H				23 (24.2%)	57 (60%)	15 (15.8%)	>0.05		
Chen	2008	China	Asian	H	277 (31.6%)	436 (49.8%)	163 (18.6%)	178 (29.1%)	308 (50.4%)	125 (20.5%)	>0.05	range 30–64	
Iwasaki^1^	2009	Japan	Asian	H	69 (17.8%)	194 (50%)	125 (32.2%)	82 (21.1%)	188 (48.5%)	118 (30.4%)	>0.05	cases 53.8 controls 54.0	cases controls pre 12% 9% post 88% 91%
Iwasaki^2^	2009	Brasil (japanese)	Asian	H	13 (16.5%)	44 (55.7%)	22 (27.8%)	14 (17.7%)	41 (51.9%)	24 (30.4%)	>0.05	cases 56.6 controls 56.5	cases controls pre 8% 8% post 92% 92%
Iwasaki^3^	2009	Brasil	Caucasian	H	58 (15.3%)	200 (52.8%)	121 (31.9%)	67 (17.7%)	179 (47.2%)	133 (35.1%)	>0.05	cases 52.4 controls 52.5	cases controls pre 11% 10% post 89% 90%
Pineda	2012	Spain	Caucasian	H	311 (23.1%)	629 (51.5%)	281 (25.4%)	109 (20.9%)	278 (53.3%)	135 (25.9%)	0.094	cases 51 (21–89) controls 51 (18–86)	cases controls TT CT CC TT CT CC pre 22% 59% 19% 25% 53% 22% post 21% 50% 29% 26% 51% 23%

### Meta-analysis Results

When the eligible studies were pooled in the meta-analysis, no significant association with breast cancer risk was found in any of the genetic models. The dominant model did not present homogeneity when either the fixed effect model or the random effect model were applied. We assessed an analysis of influence to discriminate if one of the studies was causing the heterogeneity. The Yoshimoto et al. [28] study shows the highest p-value for the heterogeneity test (p = 0.16), revealing it as the more discordant of all in the comparison. Consequently, we eliminated this study from further analysis. Additionally, the fixed effect model did not show homogeneity for two of the assayed models. To explore whether the sources of heterogeneity were mean age and menopausal status, we performed a meta-regression analysis. Neither of these variables explained the heterogeneity (F = 3.042; p = 0.247). The random effect model was chosen for all the analyses.

**Figure 2:**
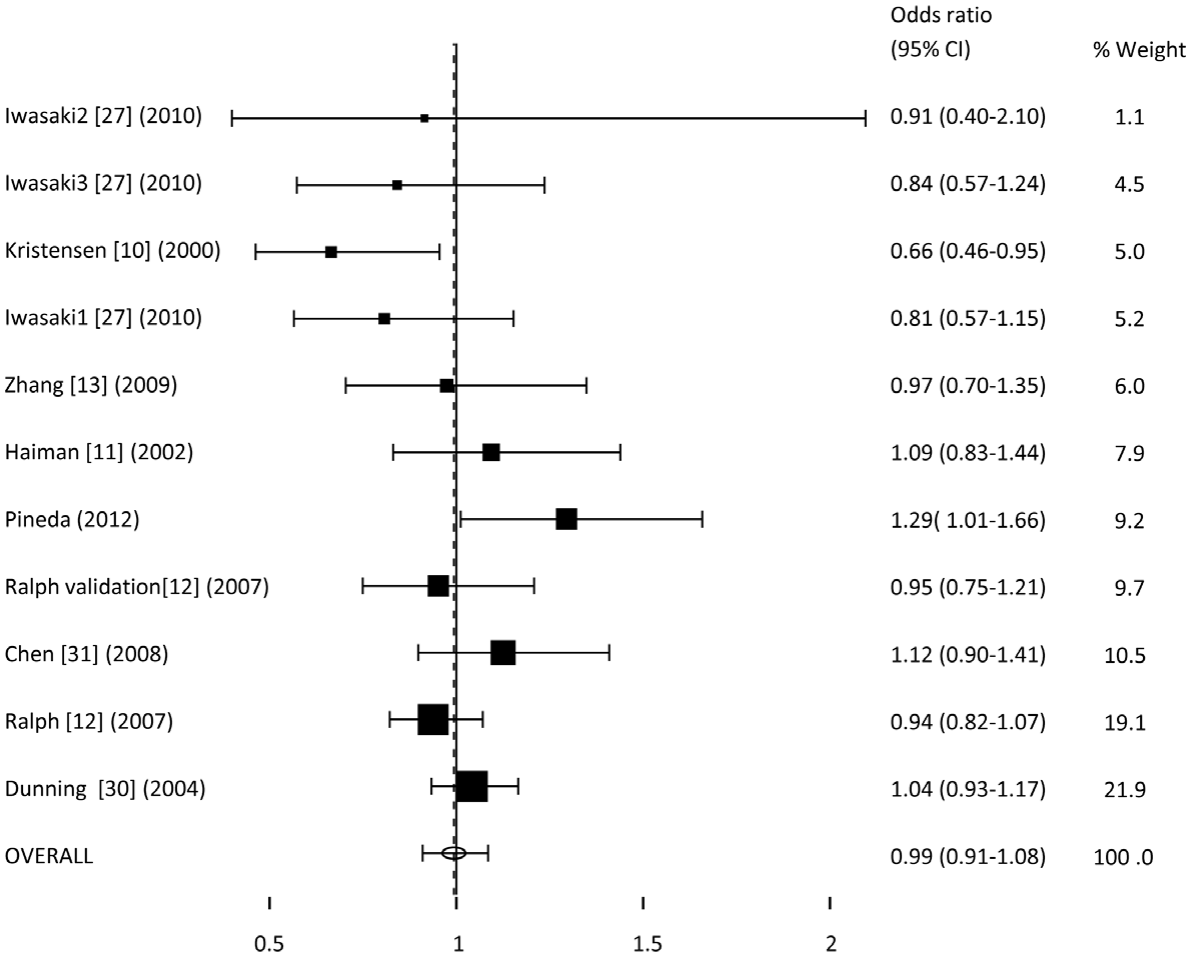
Meta-analysis of OR for rs10046 polymorphism associated with breast cancer (dominant model).

For the dominant model, the odds ratio obtained was 0.99 (95% CI; 0.91–1.08) (Figure 2). This result shows no association with the presence of the C allele and a predisposition to breast cancer.

**Figure 3:**
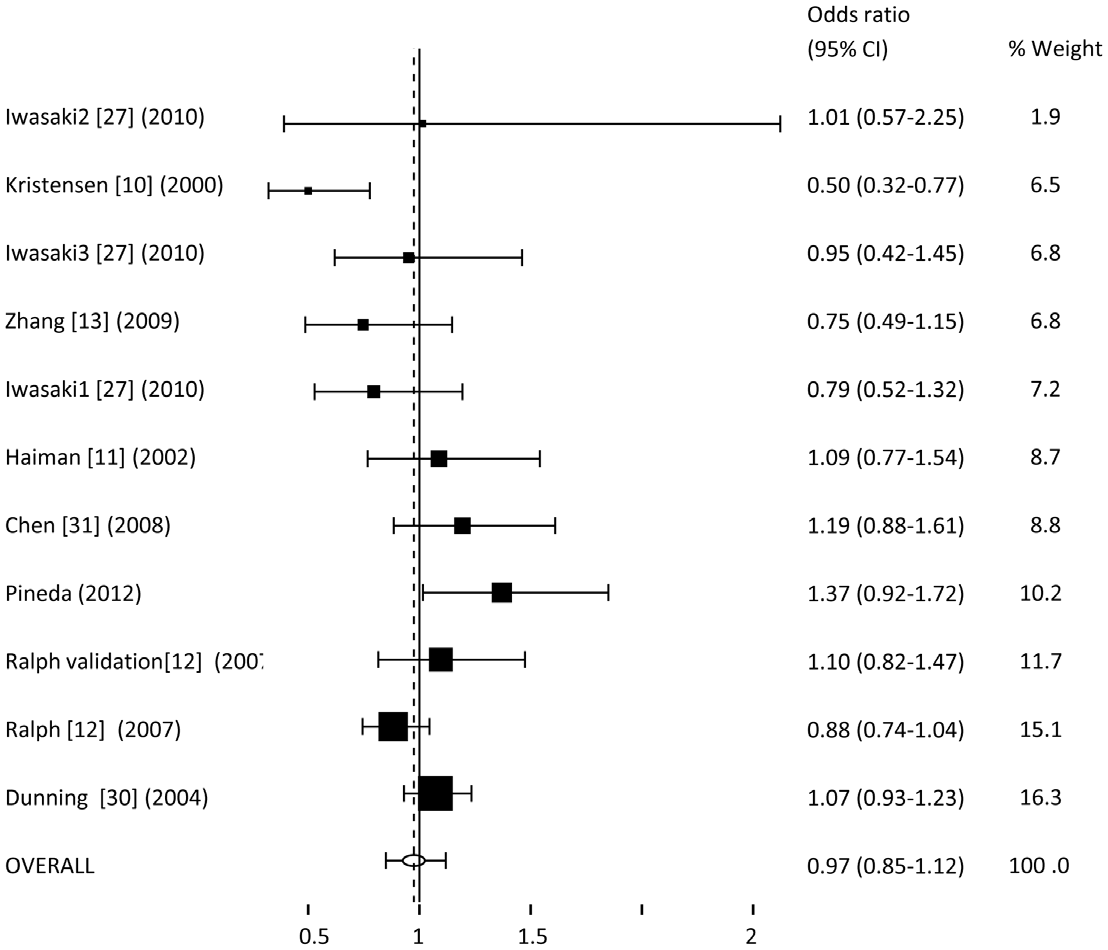
Meta-analysis of OR for rs10046 polymorphism associated with breast cancer (CT vs.TT).

**Figure 4:**
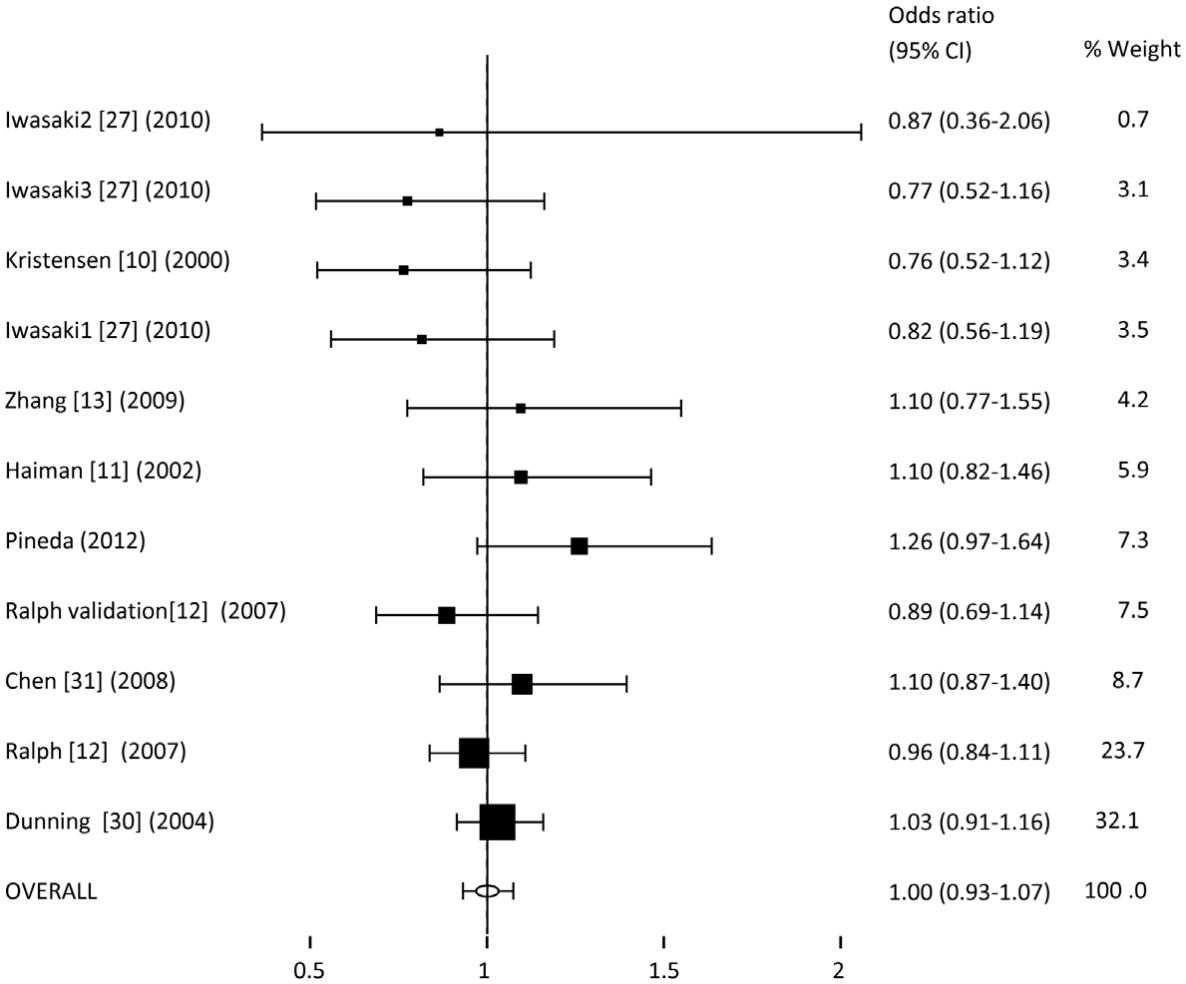
Meta-analysis of OR for rs10046 polymorphism associated with breast cancer (CC vs. TT).

Similarly, we proceeded to evaluate the possible association using the co-dominant (Figures 3 and 4), recessive (supplementary Figure 1), additive (supplementary Figure 2) and over-dominant models (supplementary Figure 3). In all cases, the analysis presents an OR, again, nearly 1, and the 95% CI crossed this limit. We can conclude that neither of the models presents an association with the risk to develop breast cancer for the rs10046.

### Subgroup Analysis

Considering the possible impact of ethnic variations, we then performed two subgroup analyses in European, Asian and American populations, and in Caucasian, Asian and others ethnicities. The analyses of these groups failed to suggest an association between rs10046 and breast cancer risk in any of the models (Table 3).

**Table 3:** Association between rs10046 and breast cancer risk stratified by subgroups.

GENETIC MODEL													
Subgroup A	n	RECESSIVE		DOMINANT		ADDITIVE		CC vs. TT		CT vs. TT		OVERDOMINANT	
		OR	95% CI	OR	95% CI	OR	95% CI	OR	95% CI	OR	95% CI	OR	95% CI
0	4	1.03	0.89–1.18	0.95	0.86–1.06	0.95	0.86–1.05	0.95	0.84–1.08	0.95	0.85–1.06	0.95	0.84–1.07
1	3	0.94	0.70–1.26	1.00	0.76–1.32	0.98	0.72–1.34	0.94	0.61–1.44	1.03	0.84–1.27	1.02	0.93–1.11
2	4	1.08	0.79–1.48	1.01	0.86–1.18	0.99	0.84–1.17	0.94	0.72–1.21	1.02	0.86–1.21	1.15	0.91–1.44
**GENETIC MODEL**													
**Subgroup B**	**n**	**RECESSIVE**		**DOMINANT**		**ADDITIVE**		**CC vs. TT**		**CT vs. TT**		**OVERDOMINANT**	
		**OR**	**95% CI**	**OR**	**95% CI**	**OR**	**95% CI**	**OR**	**95% CI**	**OR**	**95% CI**	**OR**	**95% CI**
0	6	0.99	0.86–1.14	1.00	0.89–1.13	1.00	0.87–1.14	0.99	0.82–1.20	1.01	0.91–1.11	1.00	0.94–1.07
1	4	1.08	0.79–1.48	1.01	0.86–1.18	0.99	0.84–1.17	0.94	0.72–1.21	1.02	0.86–1.21	1.15	0.91–1.44
2	1	1.15	0.85–1.56	0.84	0.57–1.24	0.87	0.60–1.27	0.95	0.62–1.46	0.77	0.52–1.16	0.80	0.60–1.07

Subgroup A corresponds to American (0: Haiman, Ralph, Ralph validation, Iwasaki3), European (1: Kristensen, Dunning, Pineda) and Asian (2: Chen, Iwasaki1, Iwasaki2, Zhang) populations. Subgroup B corresponds to Caucasian (0: Haiman, Ralph, Ralph validation, Kristensen, Dunning, Pineda), Asian (1: Chen, Iwasaki1, Iwasaki2, Zhang) and other ethnicities (2: Iwasaki3). OR: odds ratio, CI: confidence interval. The Random effect model was used to perform the analysis.

### Evaluation of Reliability

Heterogeneity is a potential problem when performing a meta-analysis. In the present study, we systematically performed an evaluation of the heterogeneity in each model using the Q test and I^2^ statistic. The breach of the homogeneity condition led to the use of a less restrictive model (random effect model instead of fixed effect model). When the analysis was not homogeneous even using the random effect model we performed an influence analysis to discriminate study outliers and a meta-regression to explore whether the source of heterogeneity was the mean age and menopausal status variables. An influent study was detected as the source of heterogeneity and was eliminated from further analysis.

Another limitation of meta-analysis is publication bias. To check for potential problems, we performed two statistical analyses: Begg’s and Eggeŕs. The Begǵs method checks the correlation between an effect and its variability. This analysis calculates the Kendall’s tau (τ) coefficient. The absence of statistical significance suggests no publication bias. Of the three models assayed, only the recessive model showed significance (p-values = 0.14, 0.02 and 0.89 for dominant, recessive and over-dominant models, respectively). To confirm these results, we analyzed the data with the more sensitive Egger’s testwhich uses a linear regression between the reduced measure of the effect and the precision. The existence of bias was evaluated by the significance of the ordinate value in the origin for a value p<0.1. This analysis confirmed there was no publication bias (p-values = 0.40, 0.52, 0.94 for dominant, recessive and over-dominant models, respectively).

Using the Gleser-Olkin method, we estimated the number of unpublished studies based on the number of known publications and their p-values. The low limit of the 95% CI was a negative value that was compatible with the hypothesis of absence of publication bias. Furthermore, we performed a cumulative meta-analysis for year of publication to verify the influence of time on the results observed by different groups. There were no tendencies related to time that might affect the data published (data not shown).

## Discussion

The importance of breast cancer worldwide has led to a substantial increase in research in this field. The present efforts to fight the illness are focused in its better classification [29] and treatment, but without forgetting the relevance of prevention and early diagnosis. Among the factors to take into consideration in the early detection of breast cancer is the exposure to estrogens and to other hormones. Prospective studies have shown a direct association between circulating sex hormones with the risk of developing breast cancer in postmenopausal women [30]. Furthermore, the circulating levels of estradiol precursors and metabolites have also been related to increased risk of breast cancer. These circulating levels are largely under genetic control and, consequently, can be modified by polymorphisms on genes associated with estradiol. Therefore, it is logical to think that changes in genes that control the levels of estrogen, as in the case of rs10046 (*CYP19*), are potential candidates to predispose for this illness (30).

To date, several studies have been performed to evaluate this hypothesis in different populations, with contradictory results. Consequently, we have done a case-control study in a Spanish population with samples paired by age and menopausal status to assure that these variables have not affected the results. We obtained a significant association between carrying at least one C allele (dominant model) and the risk of breast cancer. This result was in agreement with previous works published, where the frequency of the C allele is higher in cases vs. controls [11], [30], [31]. Especially relevant is the publication of Dunning et al. [30], where the study was carried out in more than 2000 cases and 3000 controls. Other authors however, have not detected a significant association or even found opposite results. Whereas Kristensen et al. found a predisposition role for the T allele [10], other authors have not found a clear association with breast cancer risk [12], [13], [27]. The reason for these conflicting results could be that the studies were developed in different populations, geographical areas and, additionally, with a variable number of samples. Though some of these cases-control studies did not reach statistical significance, it is possible that the polymorphism contributed to levels of circulating sex hormones. At least some publications claim that this polymorphism is related to the levels of estradiol and the estradiol:testosterone ratio in normal postmenopausal women [30], a factor relevant in the development of breast cancer [6]. Unfortunately, in our case-control study, serum samples were not available for the determination of estradiol, which would have added important information. Published expression data show higher estrogen levels with higher repeats in [TTTA]n polymorphism on *CYP19* found in linkage disequilibrium with rs10046 [32], [33], [34]. Additionally, a highly significant relationship between aromatase SNPs and circulating estrogen levels among postmenopausal women has been found by Haiman et al. [35]. Between the highly correlated tagging SNPs, there were polymorphisms in different haplotype blocks, including rs10046.

How this SNP can affect estrogen levels is not obvious. Different studies have shown SNPs that can affect phenotypic outcome by altering DNA binding sites [36], [37], [38], mRNA stabilization, folding, splicing [39], [40], [41] and modification of mechanisms involving the enhancement of transcription and the posttranslational regulation.

All the above highlights the importance of studying in depth the possible association of C>T rs10046 SNP with breast cancer risk. With that in mind, we have performed a meta-analysis, a reliable analytical tool for comparing the different data related to this polymorphism. The results of our meta-analysis showed no significant association with breast cancer risk in any of the genetic models tested. In all cases, the overall OR calculated is near the value of 1, indicating no existence of a trend or predilection for the rs10046 genetic variants between cases and controls. They were no significant differences in age and menopausal status across genotypes, as the meta-regression analysis revealed. The comparisons of subgroups based on population stratifications showed only slight discrepancies, data that in no case reached significance.

The results of our work show that the case-control studies restricted to a limited population provide different results than do those with a wide representation of the population. To extrapolate results and to come to relevant conclusions of the possible influence of a factor in a disease seems essential to realize global studies. The meta-analysis approach allows us to obtain relevant conclusions and simultaneously to summarize and to unify the studies in the field. On the basis of our results, polymorphism rs10046 is not capable of modifying the risk to develop breast cancer. Nonetheless, several studies associated this polymorphism to circulating hormonal levels [30]. Additionally, rs10046 has been related to altered disease free survival in the subgroup of pre-menopausal breast cancer patients [9]. Polymorphisms in linkage disequilibrium with rs10046, as in the case of rs4664, rs700518 and rs700519, has been associated with variable efficacy of treatments [23], [24], [42] and breast cancer survival [43], [44].

The data available is not sufficient to affirm that it is an activating polymorphism, but published data suggest that it could be related to an advantage in the protein structure that makes it more active [23]. In the absence of a mechanistic explanation, however, strong linkage disequilibrium with other polymorphisms remains possible. There are some studies about polymorphisms in linkage disequilibrium with rs10046 which describe a significant association with efficacy of the aromatase inhibitor letrozole in patients with breast cancer, as is the case for rs700158 [42] and rs4646 [23], [24]. The latter SNP has also been reported to be associated with HER2 status of tumors [9], circulating steroid hormones [35] and histological grade and tumor size in postmenopausal women [35], [43]. Haiman et al. reported a significant relationship between this SNP and circulating estrogen levels among postmenopausal women. A haplotype analysis has also been performed on the *CYP19* gene in most of these studies. Some specific haplotypes, including rs10046, were associated with an increased risk of breast cancer with concurrent proliferative fibrocystic conditions [31] and with clinical efficacy of letrozole [42]. Moreover, the haplotype studies conducted by Raskin et al. showed a trend to association with breast cancer risk in BRCA1 carriers aged <50 years [26].

In our study, we have not done any analysis of polymorphism in linkage disequilibrium with rs10046. This could be a limitation, as it could have provided more information on the role of *CYP19* in breast cancer. Nevertheless, it should be noted that these studies were taken into account when we performed the meta-analysis even though some of them were excluded from the final statistical analysis due to lack of necessary data.

In conclusion, despite the limitations, the results of the present meta-analysis suggest that rs10046, by itself, does not directly affect the risk to suffer breast cancer. Further extensive studies to clarify the influence of *CYP19* polymorphisms on estradiol circulation levels are necessary.

## Supporting Information

Figure S1 **Meta-analysis of OR for rs10046 polymorphism associated with breast cancer (recessive model).**
(TIF)

Figure S2 **Meta-analysis of OR for rs10046 polymorphism associated with breast cancer (additive model).**
(TIF)

Figure S3 **Meta-analysis of OR for rs10046 polymorphism associated with breast cancer (over-dominant model).**
(TIF)
