# Supplementary figures and images for: Associations between Aromatase CYP19 rs10046 Polymorphism and Breast Cancer Risk: From a Case-Control to a Meta-Analysis of 20,098 Subjects
Source: PLoS One. 2013 Jan 16;8(1):e53902. doi: 10.1371/journal.pone.0053902 (PMC3547044; doi:10.1371/journal.pone.0053902)

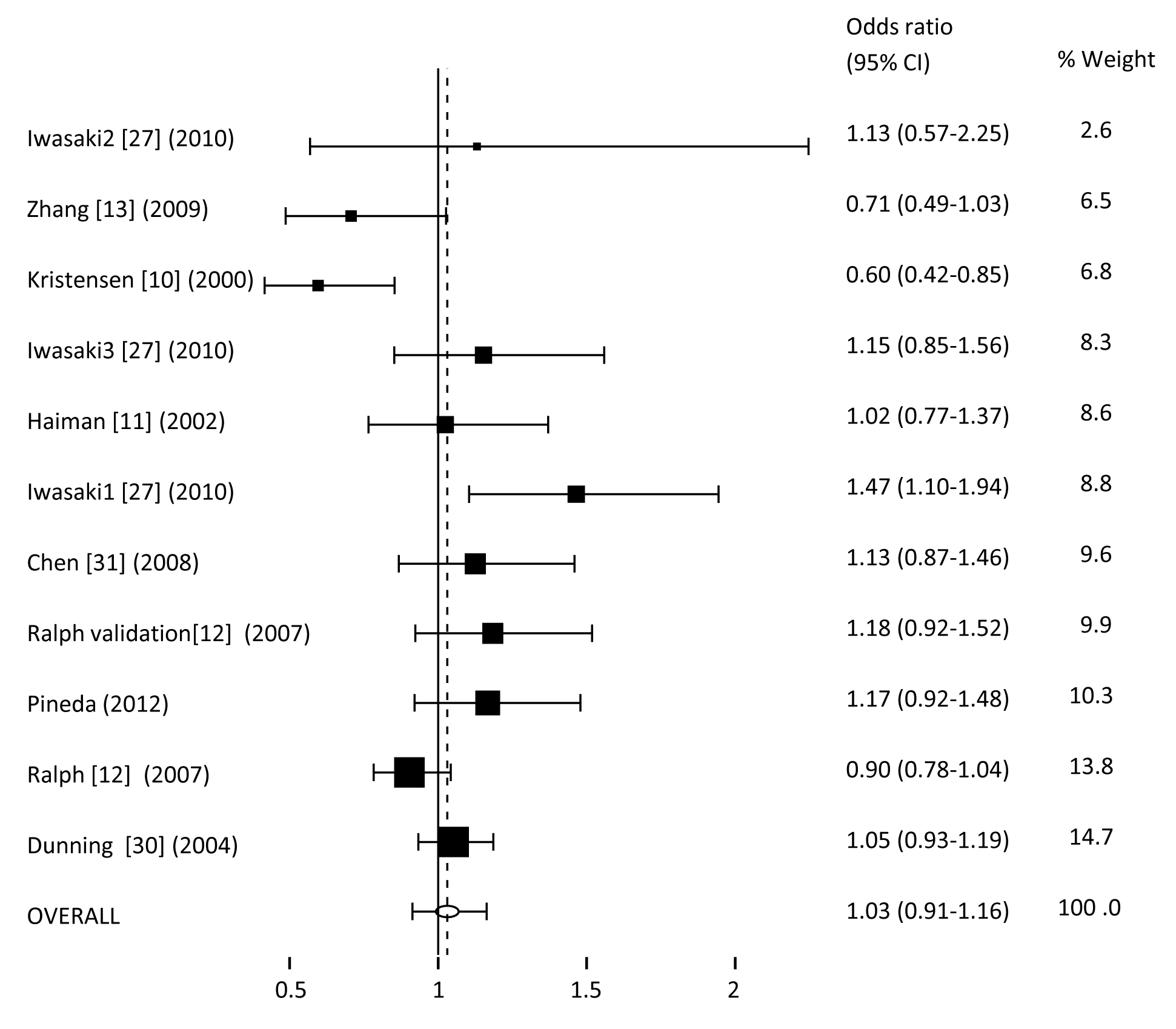

Supplement: Figure S1 — Meta-analysis of OR for rs10046 polymorphism associated with breast cancer (recessive model). (TIF) [file pone.0053902.s001.tif]

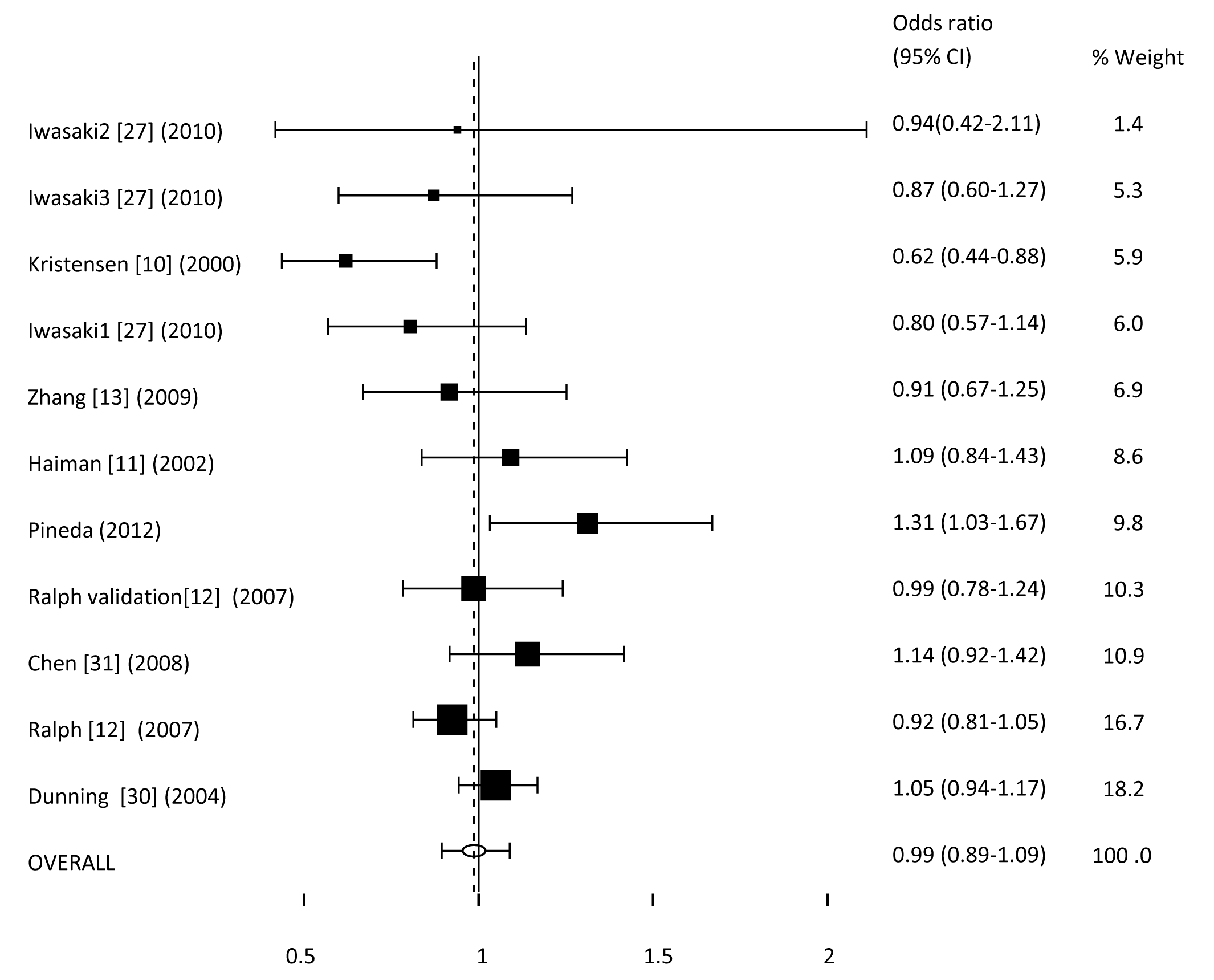

Supplement: Figure S2 — Meta-analysis of OR for rs10046 polymorphism associated with breast cancer (additive model). (TIF) [file pone.0053902.s002.tif]

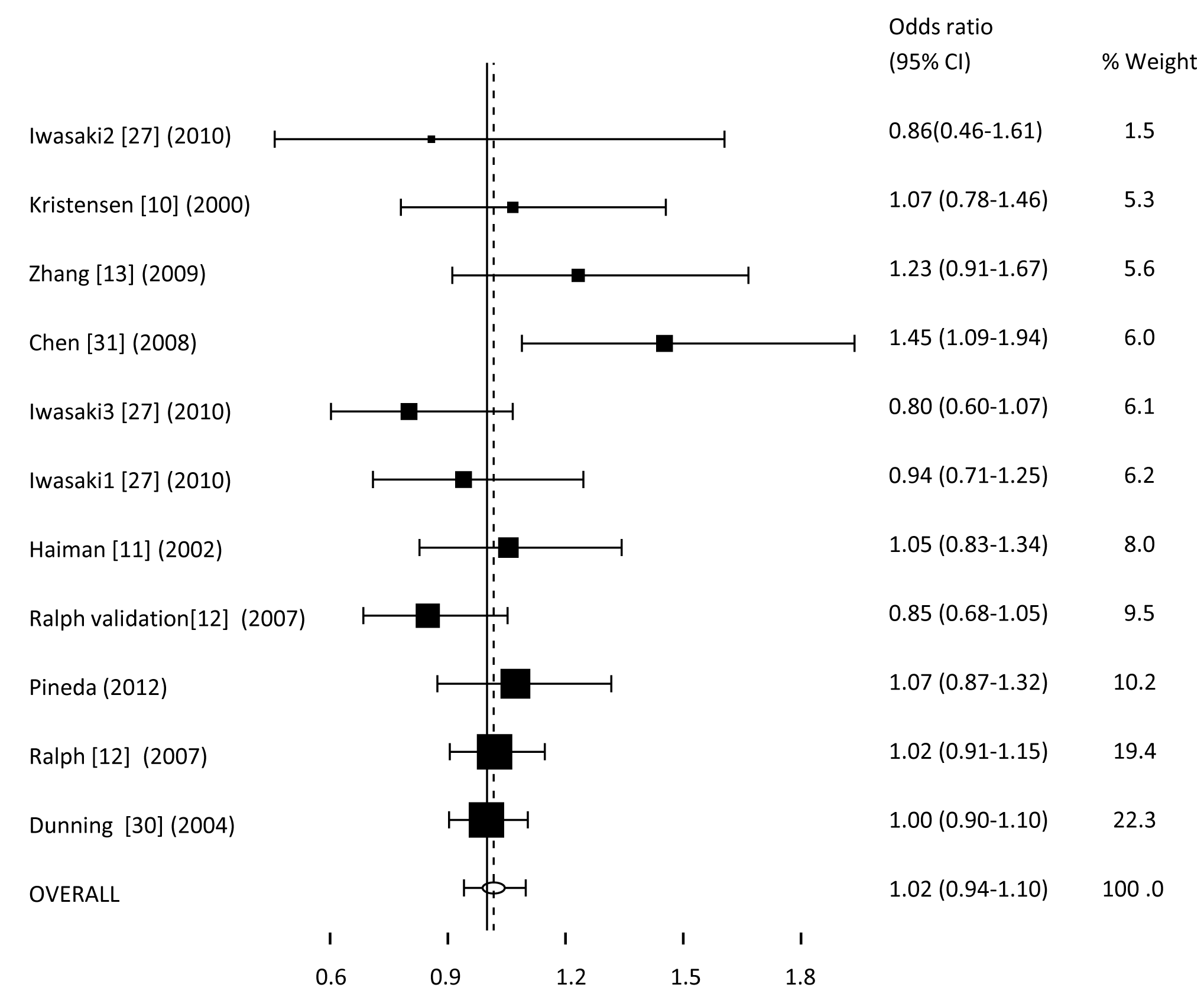

Supplement: Figure S3 — Meta-analysis of OR for rs10046 polymorphism associated with breast cancer (over-dominant model). (TIF) [file pone.0053902.s003.tif]
